# Social Media Use, Self-Efficacy, Perceived Threat, and Preventive Behavior in Times of COVID-19: Results of a Cross-Sectional Study in Pakistan

**DOI:** 10.3389/fpsyg.2021.562042

**Published:** 2021-06-17

**Authors:** Qaisar Khalid Mahmood, Sara Rizvi Jafree, Sahifa Mukhtar, Florian Fischer

**Affiliations:** ^1^Department of Sociology, International Islamic University Islamabad, Islamabad, Pakistan; ^2^Department of Sociology, Forman Christian College (A Chartered University), Lahore, Pakistan; ^3^Media and Communication Studies, International Islamic University Islamabad, Islamabad, Pakistan; ^4^Institute of Public Health, Charité – Universitätsmedizin Berlin, Berlin, Germany; ^5^Institute of Gerontological Health Services and Nursing Research, Ravensburg-Weingarten University of Applied Sciences, Weingarten, Germany

**Keywords:** coronavirus, social media use, prevention, infection management, infection control, regulation

## Abstract

Although the role of social media in infectious disease outbreaks is receiving increasing attention, little is known about the mechanisms by which social media use affects risk perception and preventive behaviors during such outbreaks. This study aims to determine whether there are any relationships between social media use, preventive behavior, perceived threat of coronavirus, self-efficacy, and socio-demographic characteristics. The data were collected from 310 respondents across Pakistan using an online cross-sectional survey. Reliability analyses were performed for all scales and structural equational modeling was used to identify the relationships between study variables. We found that: (i) social media use predicts self-efficacy (β = 0.25, *p* < 0.05) and perceived threat of coronavirus (β = 0.54, *p* < 0.05, *R*^2^ = 0.06), and (ii) preventive behavior is predicted by self-efficacy and perceived threat of coronavirus (*R* = 0.10, *p* < 0.05). Therefore, these results indicate the importance of social media's influence on health-related behaviors. These findings are valuable for health administrators, governments, policymakers, and social scientists, specifically for individuals whose situations are similar to those in Pakistan.

## Introduction

The current COVID-19 pandemic is the most significant public health crisis of this century (World Health Organization, 2020). Up to mid-May 2021, the COVID-19 pandemic has had devastating consequences, with more than 161 million confirmed cases and more than 3.35 million deaths globally (World Health Organization, 2021). In a severe public health emergency like this, people seek information from all available sources—including traditional media, interpersonal communication, and social media (Perez-Lugo, 2004). For instance, traditional media play an important role in mobilizing the community, providing authoritative information and emotional support, helping isolated people feel connected, and allocating resources (Wicke and Silver, 2009). In such circumstances, people seek information from the media in order to understand the severity of the situation, and to protect themselves (Heath and Gay, 1997). As different forms and degrees of lockdown measures were imposed to control the coronavirus outbreak (Marzouki et al., 2021), there was very limited face-to-face contact (Liu et al., 2021). Thus, people have had to rely much more heavily on social media to keep informed and stay connected (Liu, 2021). Consequently, social media usage has escalated, and it has quickly established itself as a critical medium of communication for information generation, distribution, and consumption (Effenberger et al., 2020; Fischer, 2020). Compared to conventional media (electronic and print), social media allows for quick and easy access to information, making its impact more effective than ever (Cuello-Garcia et al., 2020). Scholars have also studied the influential mechanism of health risk information on social media on individual cognition, attitudes, and actions (Lin et al., 2020).

People's perceptions of pandemic-associated risk are key factors contributing to increased public participation in disease-prevention measures (Shahin and Hussien, 2020). The majority of people around the world have heard of the coronavirus, and most of them are aware of the need to practice preventive behaviors in order to reduce its spread (Balkhi et al., 2020). Although some people follow the rules strictly, others neglect or postpone them and congregate in large groups in public areas or in their homes (Nofal et al., 2020). The fact that people behave so differently during times of collective action suggests that their perceptions of the threat posed by this virus vary greatly depending on where they live and who they are (Zhang et al., 2020).

According to Kaplan and Haenlein (2010), social media is a term that refers to a variety of applications, such as social networking sites and blogs, that are built on web 2.0 (e.g., Facebook, YouTube, and Twitter) and enable users to create, share, and engage in various activities. The term “social media” is a catch-all term for websites that offer a variety of social activities. Social networking is a web-based, electronic-mediated platform that allows users to create profiles and exchange thoughts, images/clips, and information in a virtual network system.

Previous studies have shown that people have preferred social media platforms over traditional media to obtain disease-related information in recent infectious disease outbreaks (Jang and Baek, 2019). During the H1N1 outbreak in 2009, people relied on Twitter (Chew and Eysenbach, 2010) and Facebook groups (Davies, 2009) for the exchange of information, opinions, and experiences. The public was also somewhat dependent on social media platforms to access and share MERS-related information in 2015 (Jang and Baek, 2019). But in the case of the ongoing COVID-19 pandemic, social media use has reached unprecedented levels compared to the pre-pandemic period. People may be using social media during corona-led social distancing for stress relief and with the aim of accessing entertaining content, such as movies, comedies, and communication with family and friends (Whiting and Williams, 2013). As work and schooling have been transferred to an online-based format, people are also spending a lot of time using social media to meet their professional and educational needs (Prem et al., 2020). However, social media has become more significant as these platforms have emerged as a useful medium to disseminate health messages and contribute to the betterment of psycho-behavioral responses to COVID-19.

Studies indicate that people primarily use social media, rather than other media outlets, to access information related to the coronavirus pandemic. As a result, social media platforms have been utilized for maintaining quarantine, alerting the public about high-risk areas, and providing awareness about health maintenance and treatments (Chan et al., 2020). Moreover, empirical evidence shows that the use of social media as an information-seeking platform has altered preventive behavior related to coronavirus. The results of these studies are consistent with previous research on the function of social media in improving health-related preventive behaviors during pandemic situations (Shi and Smith, 2016; Yoo et al., 2016). However, more scientific research is needed to explore the critical role of social media in coronavirus prevention and treatment. Since individual disease prevention behavior is the only known way to avoid the spread of COVID-19 (Ning et al., 2020), it is critical to recognize the factors, along with social media use, that motivate individuals to participate in disease prevention behavior. This study aims to examine how social media has played an essential role in formulating preventive behavior during the COVID-19 outbreak in Pakistan.

### Situation in Pakistan

In Pakistan, there are currently over 46 million social media users. Between 2020 and 2021, this number increased by 9.0 million (+24%) (Kemp, 2021). After the start of the coronavirus pandemic and its related measures of social isolation, the number is estimated to have risen dramatically. The coronavirus pandemic hit Pakistan in February 2020, and social distancing started to be implemented across the country in mid-March 2020 (Mahmood et al., 2020). Social media has been one of the main outlets providing news and information about the coronavirus and attempts at prevention due to social distancing (Nazir et al., 2020). Even before the outbreak of COVID-19, social media was acknowledged for its value in disseminating information about basic health awareness, health literacy, hygiene, sanitation, and nutrition (Nisar and Shafiq, 2019; Zakar et al., 2021).

The government of Pakistan released a new social media regulation policy in January 2020, but this policy still fails to include social media's role in health risk communication and health literacy. Due to the outbreak of coronavirus, the government decided to review the country's social media regulations in order to maximize its potential for improving health literacy among the general population. In this pandemic situation, the government should use social media to provide therapy to people in order to improve their mental health and coping skills (Nisar and Shafiq, 2019). As a result, empirical research on the role of social media in encouraging potentially protective and health-seeking behavior is needed.

### Theoretical Framework

In order to understand infectious disease outbreaks, a systematic review of the literature was carried out (Yang, 2015). It was found that predicting preventive behaviors during infectious disease outbreaks has been the focus of many studies. Furthermore, most of these studies used the extended parallel process model (EPPM) (Witte, 1992, 1994) in order to understand how individuals experience and respond to an infectious disease. The EPPM has been used in previous empirical studies conducted on preventive behaviors during infectious disease outbreaks (Siu, 2008; Balicer et al., 2010; Zhang et al., 2015) and, more specifically, in studies showing the relationship between social media usage and preventive behavior during pandemics (Zhang et al., 2015; Shi and Smith, 2016).

**Figure 1:**
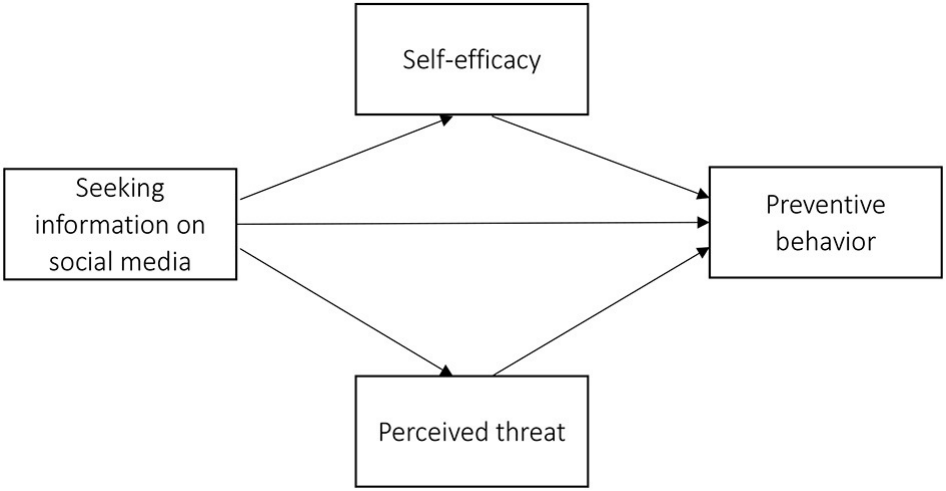
Conceptual model of the study based on the theory of EPPM.

This study also uses the EPPM as its conceptual background (Figure 1). This model develops the significance of rational considerations and emotional reactions in determining health-related behavioral decisions (Witte, 1992, 1994). The degree to which a person feels threatened by a health issue determines his or her motivation to act, while one's self-efficacy or confidence to effectively reduce the threat determines the action itself. In other words, self-efficacy and the perceived level of threat of any disease influence the extent to which people opt for preventive behavior (Witte, 1992, 1994). When both self-efficacy and perceived threat are high, individuals are likely to employ recommended preventive behaviors in order to avoid the danger (Yoo et al., 2016).

Perceived threat refers to the subjective evaluation of the threat contained in the message. It is a cognitive construct that comprises two dimensions: perceived severity of the threat and one's perceived susceptibility to that threat (Popova, 2012). Perceived severity is defined as “one's feelings concerning the seriousness” of a threatening event (Gore and Bracken, 2005), whereas perceived susceptibility refers to beliefs about the probability of personally experiencing the threat (Witte et al., 1998). In the EPPM, self-efficacy is defined as beliefs about one's ability to carry out the recommended response (Witte, 1996). Individuals' beliefs about their capabilities influence their behavior, such as what they choose to do or how they respond, in order to effectively manage situations (Bandura, 1997). Based on the EPPM, the existing literature affirms the relationship between self-efficacy and preventive behavior in relation to infectious disease. Similarly, the perceived threat of any infectious disease leads to the adoption of preventive behavior related to that infectious disease.

### Research Hypothesis

The following research hypotheses were made:

1. There is a positive relationship between social media use and self-efficacy among the respondents.
2. There is a positive relationship between social media use and perceived threat of COVID-19 among the respondents.
3. Social media use predicts the preventive behavior related to COVID-19 among the respondents.
4. Perceived threat of COVID-19 explains the preventive behavior related to COVID-19 among the respondents.
5. Self-efficacy explains the preventive behavior related to COVID-19 among the respondents.

## Methods

### Data Collection

In these days of social distancing and lockdown, we opted for an online survey using Google Survey. Participants were recruited via announcements published on social media (Facebook, LinkedIn, and WhatsApp). A link to the questionnaire was also posted on the social media pages of various universities in Pakistan. Data was collected during the period April 10–30, 2020. For this study, the age limit was set at 18 years and above. Respondents were briefed about the objectives of the study by means of a cover letter, and informed consent was taken electronically. Participation in the study was voluntary and no incentives were provided to respondents. Each question in the questionnaire was compulsory and the respondent could not submit the form without answering all the questions. If any respondent did not want to answer all the questions, he or she was allowed to exit the survey. Overall, 310 respondents successfully completed the survey. The questionnaire was administered in English, as this is the official working and study language in Pakistan (Supplementary Appendix 1).

### Measures

#### Sociodemographic Characteristics

Information about age, gender, provincial belonging, area of residence (urban vs. rural), marital status, monthly family income, and occupation of the respondents were collected in order to control for the effects of socio-demographic characteristics on social media use, self-efficacy, perceived threat, and preventive behavior.

#### Perceived Threat of COVID-19

In order to develop the scale to measure perceived threat of COVID-19, the authors reviewed the existing scales that have previously been developed to assess perceived threat during infectious disease outbreaks. For instance, a scale was developed by Yang (2015) to assess the perceived threat of H1N1. Similarly, a scale was derived from it to measure the perceived threat of MERS (Yoo et al., 2016). In this study, the authors modified this scale to make it suitable for measuring the perceived threat of COVID-19. To measure perceived susceptibility, three items were used (“COVID-19 could happen to me,” “it could happen my family,” and “it could happen to my neighbors and friends”). Perceived severity was measured with four items [“COVID-19 causes death quickly,” “Many people can die from COVID-19,” “A person who contracts COVID-19 will die if not treated,” and “COVID-19 is fatal,” strongly disagree (1) to strongly agree (5)].

#### Self-Efficacy

In earlier studies, a four-item scale was used to measure self-efficacy for MERS (Yoo et al., 2016). The authors modified this scale to measure self-efficacy for COVID-19 using following four items (“I can figure out how to avoid COVID-19 infection,” “I can avoid COVID-19 infection,” “I can recover even if I contract COVID-19,” and “I am fully informed about COVID-19”). A five-point Likert scale, strongly disagree (1) to strongly agree (5), was used for this scale.

#### Preventive Behavior

In order to control outbreaks of infectious diseases like COVID-19, health experts and global health agencies [e.g., the World Health Organization (WHO)] recommend a series of preventive behaviors, such as hand hygiene, and avoidance behaviors, such as social distancing or (voluntary) quarantine (Karimi et al., 2015; Weston et al., 2018; Lewnard and Lo, 2020). The authors developed a preventive behavior scale consisting of three constructs (handwashing, cough etiquette, and social distancing behavior). This scale was constructed using the guidelines for COVID-19 prevention recommended by the WHO. Handwashing behavior was measured with five items (using hand sanitizer, washing hands before making and eating food, and washing hands whenever they feel dirty and after using the bathroom). Cough etiquette behavior was assessed with three items (covering the mouth and nose while sneezing, coughing or sneezing into the arm if having no tissue, putting the used tissue into a covered dustbin). In measuring social distancing behavior, five items were used (avoiding shaking hands with people, maintaining social distancing when going outside, avoiding going out unnecessarily or visiting sick people, and not touching body parts). A five-point Likert scale, not at all (1) to always (5), was used for this scale.

#### Social Media Use

Social media usage during COVID-19 was measured using two constructs: for medical information (related to COVID-19) and for general information. Retrieving or sharing general information on social media included: homebased remedies and the names of herbal medicines useful for boosting immunity in response to COVID-19, the names of tablets or injections being used for the treatment of COVID-19, and religious texts for protection from sickness and ailments. Five items were used to measure medical information received or shared using social media [appropriate techniques for wearing a face mask, the availability of hand sanitizer and face masks, consulting doctors if feeling unwell, and keeping oneself updated on the situation of the pandemic; strongly disagree (1) to strongly agree (5)].

### Reliability Analysis

The reliability analysis revealed that the scales used to measure the study variables (perceived threat of coronavirus, preventive behavior, social media use, and self-efficacy) were highly reliable in the Pakistani context. The values of Cronbach's Alpha for perceived susceptibility and perceived severity were 0.815, and 0.762, respectively. The scales used for preventive behavior also showed high reliability. Cronbach's Alpha was 0.751 for handwashing, 0.708 for cough etiquette, and 0.830 for social distancing behavior. The reliability was higher for the scale of medical social media use (0.823) than for general use (0.622). For self-efficacy, it was also satisfactory, at 0.700 (Table 1).

**Table 1:** Psychometric properties of the study variables.

**Scales**	**α**	**Mean**	**SD**	**Min**.	**Max**.	**Number of items**
**Perceived threat related to COVID-19**						
Perceived susceptibility	0.815	13.44	9.626	4	20	4
Perceived severity	0.762	13.37	9.715	4	20	4
**Preventive behavior**						
Handwashing	0.751	22.19	10.543	6	25	5
Cough etiquette	0.708	13.09	5.312	3	15	3
Social distancing behavior	0.830	21.54	3.713	5	25	5
**Social media use**						
Medical use	0.823	15.69	17.386	5	25	5
General use	0.622	13.80	9.748	4	20	4
**Self-efficacy**	0.700	14.85	2.64	4	20	4

### Data Analysis

The data analysis was conducted using SPSS Amos. We derived frequencies and percentages in order to describe the sociodemographic characteristics of study participants. *T*-tests were used to check differences between genders, social media use, perceived threats, and preventive behavior. The Pearson correlation coefficient was calculated to investigate the roles of age and income in explaining perceived threats related to the coronavirus, preventive behavior, and social media use. Based upon previous research conducted to predict preventive behavior during infectious disease outbreaks (Yoo et al., 2016), we performed structural equation modeling (SEM). We combined exploratory factor analysis and multiple regression as a confirmatory technique to investigate the relationship between the dependent variable (preventive behavior), independent variable (social media use), and mediating variables (perceived threat related to the coronavirus and self-efficacy). The significance level was assigned at 95% for all tests.

## Results

### Sociodemographic Characteristics

Table 2 presents the sociodemographic characteristics of the sample. Of the 310 respondents, slightly more than half were women (54.2%, *n* = 168) and the majority were unmarried (72.3%, *n* = 224). More than half of the participants were students (56.1%, *n* = 174), leading to an overall young sample. The majority had a combined family income of less than or equal to PKR 100,000 (74.5%, *n* = 231). A considerable number of respondents used social media for more than 4 h a day (42.3%, *n* = 131), and more than half of the participants (59.4%, *n* = 184) relied on social media for information about COVID-19.

**Table 2:** Sociodemographic characteristics (*n* = 310).

**Variables**	***n***	**%**
**Gender**		
Female	168	54.2
Male	142	45.8
**Marital status**		
Single	224	72.3
Married	82	26.5
Divorced/widowed	4	1.3
**Occupation**		
Employed	85	27.4
Unemployed	30	9.7
Self-employed/housewife	21	6.8
Student	174	56.1
**Age**		
18–20 years	74	23.9
21–30 years	176	56.8
31–40 years	47	15.2
41–50 years	7	2.3
>50 years	6	1.9
**Monthly family income**		
≤ PKR 100,000	231	74.5
PKR 10,0001 to 200,000	54	17.4
>PKR 200,000	25	8.1
**Daily social media use**		
<1 h a day	29	9.4
1–2 h a day	65	21.0
3–4 h a day	85	27.4
>4 h a day	131	42.3
**Relying on social media for information during COVID-19**		
Yes	184	59.4
No	90	29.0
At times	36	11.6

### Gender Differences

We conducted an independent sample *t*-test to determine the effect of gender. Overall, there was no statistically significant effect of gender on perceived threat related to the coronavirus or social media use among the respondents, except for a higher general use of social media among women. Furthermore, women displayed significantly better preventive behaviors on all three scales (Table 3).

**Table 3:** Independent sample *t*-test between gender and study variables.

**Variables**	**Gender**	**Mean**	**SD**	***t***	***p*-value**	**95% CI**	
						**Lower**	**Upper**
**Perceived threat related to COVID-19**							
Perceived susceptibility	Male	13.39	3.35	−0.24	0.804	−0.785	0.609
	Female	13.48	2.89				
Perceived severity	Male	13.19	2.92	−0.92	0.357	−1.027	0.372
	Female	13.52	3.27				
**Preventive behavior**							
Handwashing	Male	21.78	3.46	−2.04	0.041	−1.479	−0.029
	Female	22.54	3.02				
Cough etiquette	Male	12.59	2.59	−3.56	<0.001	−1.428	−0.413
	Female	13.51	1.94				
Social distancing	Male	21.08	4.10	−2.00	0.046	−1.673	−0.015
	Female	21.93	3.32				
**Social media use**							
Medical use	Male	15.89	4.36	0.76	0.445	−0.572	1.299
	Female	15.52	4.00				
General use	Male	14.16	3.14	−2.57	0.010	−1.530	−0.206
	Female	15.03	2.78				
**Self-efficacy**	Male	14.60	2.54	−1.84	0.068	−1.146	−0.040
	Female	15.15	2.75				

### Pearson Correlation

The Pearson correlation coefficient was computed to investigate the role of age and economic status in explaining the perceived threat of COVID-19, self-efficacy, preventive behavior, and social media use. The results reveal that there was no statistically significant association between age and preventive behavior. Respondents' age was negatively and very weakly correlated with perceived severity and social media use for medical purposes. The findings also suggest that economic status does not matter because monthly family income did not correlate with preventive behavior or social media use. Monthly family income was only correlated with perceived threat of coronavirus. This relationship was negative in nature and also very weak (Table 4).

**Table 4:** Pearson correlation analysis.

**Variables**	**Age**	**Monthly family income**
**Perceived threat related to COVID-19**		
Perceived susceptibility	−0.013	−0.002
Perceived severity	−0.122^*^	−0.077
**Preventive behavior**		
Handwashing	0.062	−0.100
Cough etiquette	−0.015	0.074
Social distancing behavior	−0.056	0.034
**Social media use**		
Medical use	−0.112^*^	−0.018
General use	0.041	0.042
**Self-efficacy**	0.042	−0.027

* *p < 0.05*.

### Structural Equation Model

Table 5 illustrates fit indices for the following model. The chi-square test (χ^2^ = 2.23, *p* < 0.05) and goodness of fit index (GFI = 0.95) demonstrated a good model fit. Moreover, alternate fit indices (CFI = 0.92, AGFI = 0.92, RMSEA = 0.06) confirmed the acceptable fit of the sample.

**Table 5:** Structural equation model fit indices.

**Model fit indices**	**Good fit**	**Acceptable fit**	**Model values**
Normed Chi square (X^2^/d)	X^2^/d <3	3 < X^2^/d <5	2.23
GFI	0.95 ≤ GFI ≤ 1	0.90 ≤ GFI ≤ 0.95	0.95
AGFI	0.95 ≤ AGFI ≤ 1	0.90 ≤ AGFI ≤ 0.95	0.92
CFI	0.95 ≤ CFI ≤ 1	0.90 ≤ CFI ≤ 0.95	0.92
RMSEA	0 < RMSEA <0.05	0.05 < RMSEA <0.08	0.06

Table 6 depicts path coefficient estimates for the observed variables loaded on three latent variables for this study. All of the coefficients between the perceived threat of coronavirus and its observed variables were found to be significant (*p* < 0.005). This result supports the assertion that the two observed variables—perceived susceptibility (β = 0.83) and perceived severity (β = 0.61)—significantly explained the perceived threat of coronavirus. Similarly, the coefficients between preventive behavior and its observed variables are also significant (*p* < 0.005). This result confirms that the three observed variables—handwashing (β = 0.61), cough etiquette (β = 0.67), and social distancing (β = 0.72)—have a significant positive effect on preventive behavior. In addition, the observed variables medical use (β = 0.88) and general use (β = 0.23) significantly load on the latent variable of social media use among the respondents.

**Table 6:** Path coefficients of observed variables.

**Latent variable**	**Observed variable**	**Path coefficients (β)**
**Perceived threat related to COVID-19**	Perceived susceptibility	0.83^***^
	Perceived severity	0.61^***^
**Preventive behavior**	Handwashing	0.61^***^
	Cough etiquette	0.67^***^
	Social distancing behavior	0.72^***^
**Social media use**	Medical use	0.88^***^
	General use	0.23^***^

*** *p < 0.001*.

**Figure 2:**
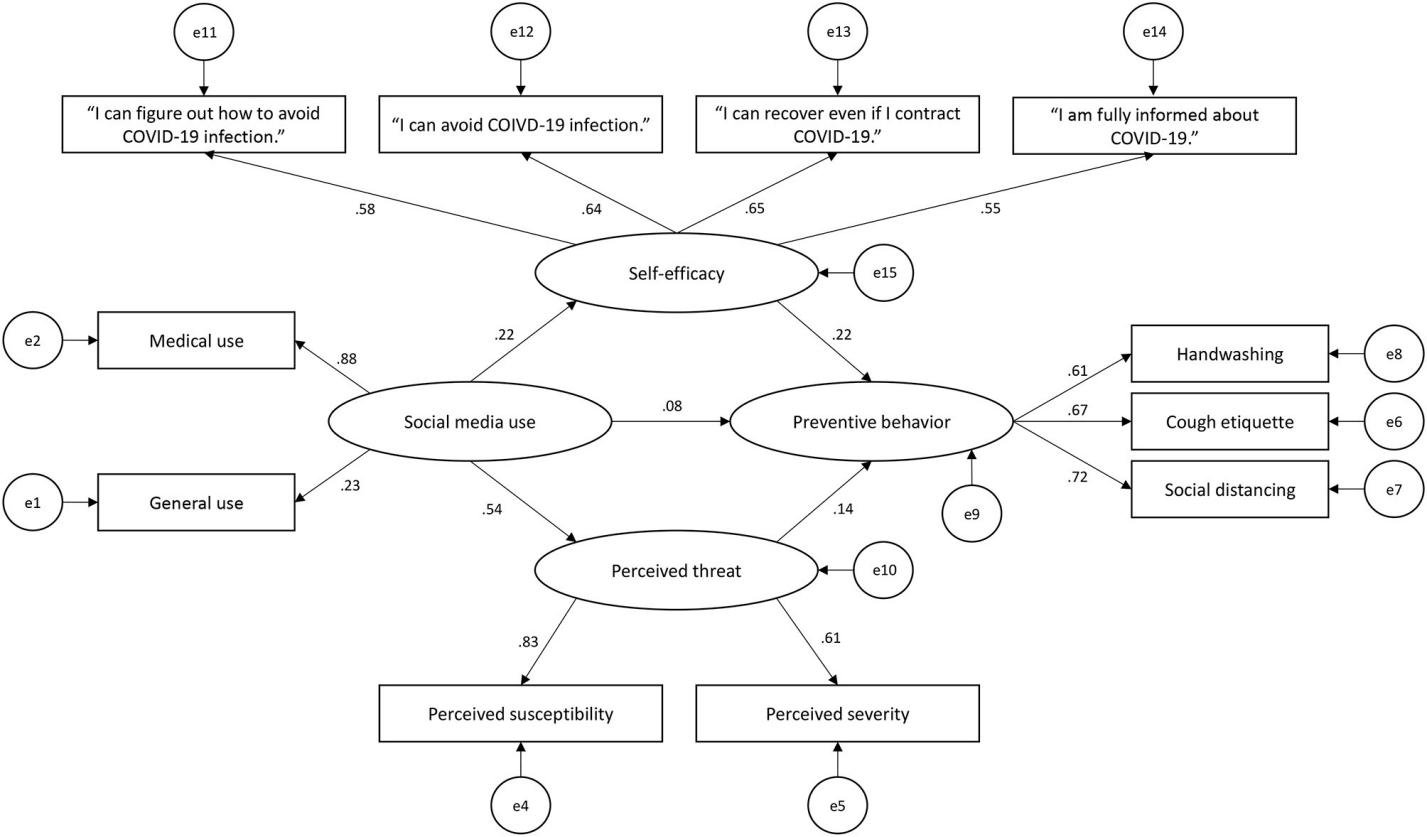
Structural equation model of social media use, self-efficacy, perceived threat related to COVID-19, and preventive behavior.

The results (Figure 2) show that there is a relationship between social media use and self-efficacy (β = 0.25, *R*^2^ = 0.06, *p* < 0.05), and social media use and perceived threat of coronavirus (β = 0.54, *p* < 0.05). In addition, perceived threat of coronavirus (β = 0.14, *p* < 0.05) and self-efficacy related to coronavirus (β = 0.22, *p* < 0.05) significantly explain preventive behavior related to coronavirus among the respondents (*R* = 0.10, *p* < 0.05).

## Discussion

The COVID-19 pandemic has permanently altered the global landscape. The crippling consequences of the lockdown have been felt in all spheres of life (Bae et al., 2021), including a crumbling health system (Miller et al., 2020; Mahmood et al., 2021), panic buying (Ahmad and Murad, 2020; Arafat et al., 2020), a severe and difficult-to-resolve economic and labor crisis (Sukharev, 2020), high levels of distress (Cullen et al., 2020), and so on. Both the short-term and long-term effects of the COVID-19 pandemic have influenced how people view and represent current events and future scenarios, including adherence to preventive behavior (Liu, 2021). This research aims to advance our understanding of how social media shapes public perceptions of threat and their involvement in preventive behaviors by analyzing data collected during the COVID-19 outbreak in Pakistan in 2020. According to the results, more than half of the participants used social media to learn about COVID-19. This finding corroborates the findings of previous research looking at how people search for and share information during epidemics (Sharma et al., 2017). People's use of social media to exchange information about their opinions and activities continues to increase in popularity (Vitak et al., 2011). Social media may be well-suited for individuals to share their opinions and views on particular health issues due to the affordances offered by social media platforms. Users of social media may find satisfaction in purposefully expressing themselves on these platforms (Fogg and Iizawa, 2008).

The unexpected and deadly COVID-19 pandemic has prompted an increasing number of studies on its effects, especially on risk perception, with the goal of providing useful information for future health-related communication strategies (Liu, 2021; Oh et al., 2021). The results show that social media use is linked to perceived threat and self-efficacy, both of which are associated with coronavirus preventive behavior. In the situation of infectious disease outbreaks, social media has grown in importance as a risk and crisis coordination tool (Strekalova, 2017; Lwin et al., 2018). Information seeking and sharing through social media can complicate disease communication, as emotions can influence both public perceptions and behavior related to infectious diseases (Apuke and Omar, 2021; Dadaczynski et al., 2021; Soroya et al., 2021). In the literature, the dynamics between social media use, affective responses, risk perception, and behavioral outcomes have been discussed (Karasneh et al., 2021). The findings revealed that people's perceptions of the COVID-19 threat were high, and that most people took self-preventive measures and believed they were helpful. This study explains the emotional and cognitive mechanisms that affect people's threat perceptions and preventive behaviors as a result of information available on social media. The researchers (Liu, 2021) also found that social media plays a role in fostering preventive behaviors by inducing fear, which influences people's risk perceptions. In China, it is reported that people were exposed to COVID-19-related information through a variety of social media platforms, which had a positive impact on preventive behaviors (Liu et al., 2021). In another study, it is concluded that the large amount of COVID-19 information made available via social media was linked to the public's understanding of their susceptibility to and the severity of COVID-19 infections, as well as their subsequent involvement in COVID-19 prevention behaviors (Lin et al., 2020).

The use of social media as a communication tool during an infectious disease epidemic is a novel form of observation, but it offers a possible source of reliable and timely assessments of disease development within populations (Nazir et al., 2020). Developing countries, such as Pakistan, usually lack the resources to sustain and monitor the surveillance system in a timely manner during an outbreak of an infectious disease (Eke, 2011). Therefore, most developing countries use social media networks as health networking mechanisms to prevent and monitor the spread of infectious disease in their communities due to a lack of funding. Social media can provide a quick method of surveillance that predicts the real-time burden of infectious disease and, as a result, can direct outbreak prevention strategies (Bhatia et al., 2021). Based on the findings of this research, the authors suggest that the government, health sector, and other stakeholders, such as media experts, collaborate to design a program for using social media platforms as health communication tools to prevent and track the spread of infectious disease in Pakistan.

## Limitations

This study has some limitations. The authors utilized social media to recruit participants only from Pakistan. Thus, while the findings may not be easily generalized to other developing countries, they are useful for governments, politicians, policymakers, health administrators, and social scientists, especially those in similar situations to Pakistan. The authors were also unable to assess other variables underlying the category of social sciences and their role in dealing with COVID-19. Future researchers could investigate other factors related to social media use, such as psychological stress, family relationships, social isolation, and loneliness. However, one of this study's strengths is that it sought to develop a local scale to assess social media usage and its relationship to perceived threat, self-efficacy, and preventive behavior, which could be used in future health communication and infectious disease management studies. To our knowledge, this is the first study of its kind conducted in Pakistan that empirically identifies the relationship between social media use, preventive behavior, self-efficacy, and perceived threat.

## Conclusion

In conclusion, social media has become an increasingly popular source of awareness and information for health communication, especially during an outbreak of disease. In an emergency, social media enhances health-risk communication by disseminating relevant information and encouraging people to engage in preventive behaviors. The current study contributes to health risk communication scholarship by using an expanded parallel process model (EPPM). This study adds to the growing body of knowledge revealing that using social media to disseminate COVID-19 information can influence audiences' perceptions of perceived threat and self-efficacy, as well as their preventive behavior. This means that social media can be used as part of public health communication during outbreaks. Official social media pages for experts and health agencies could share timely and important information with the public, potentially counteracting the negative impacts of other types of media sharing.

## Data Availability Statement

The raw data supporting the conclusions of this article will be made available by the authors, without undue reservation.

## Ethics Statement

The studies involving human participants were reviewed and approved by ethical committee from the International Islamic University Islamabad. The patients/participants provided their written informed consent to participate in this study.

## Author Contributions

QM, SJ, and SM conceptualized the study. QM and SM contributed to data collection. SJ and FF supervised the work and supported in data analysis. QM drafted the draft. All authors contributed to the article and approved the submitted version.

## Conflict of Interest

The authors declare that the research was conducted in the absence of any commercial or financial relationships that could be construed as a potential conflict of interest.
