# Supplementary material for: Social Media Use, Self-Efficacy, Perceived Threat, and Preventive Behavior in Times of COVID-19: Results of a Cross-Sectional Study in Pakistan
Source: Front Psychol. 2021 Jun 17;12:562042. doi: 10.3389/fpsyg.2021.562042 (PMC8245845; doi:10.3389/fpsyg.2021.562042)
Supplement: Supplementary file 1 [file Data_Sheet_1.PDF]

ROLE OF SOCIAL MEDIA IN PROMOTING COVID-19 PREVENTIVE BEHAVIOR IN PAKISTAN

SECTION ONE

Age:

Gender: a) Male b) Female

Education: a) Primary b) Matriculation c) Graduation d) Post-Graduate

Monthly Family Income:

Marital Status: Married Not Married Divorcee/Widow

Occupation: Employed Not Employed House Wife Student

Province: **Sindh** **Punjab** **Baluchistan** **KPK**

ICT **Gilgit Baltistan** **AJK**

Locality: a) Urban b) Sub-urban c) Rural

1. Please tick your sources of information regarding COVID-19?
   a) Social media
   b) Newspapers
   c) TV
   d) Any other _________________
2. For how long you use social media in a day?
   a) Less than an hour
   b) 1-2 hours a day
   c) 3-4 hours a day
   d) More than 4 hours a day
3. Please tick the social media platform you are using the most to access COVID-19 related information?
   a) Facebook
   b) Whatsapp
   c) Twitter
   d) Youtube
   e) Instagram
   f) Any other _______________________
4. Are you using more on social media now a days ?
   a. Yes
   b. No
5. Are you relying more on social media for information since the COVID-19 outbreak?
   a) Yes B) No

Section TWO

Receiving information related to the treatment of COVID-19

1. I receive the names of tablets or injections related to the treatment of COVID- 19 on my social media accounts.
2. I receive the names of herbal medicine to cure myself from coronavirus.
3. People send some home based remedies that can be useful for Covid-19.
4. People send me religious texts on social media for the protection from this virus.

Social Media Use related to COVID-19

1. People send meme (funny images) on my social media accounts about coronavirus.
2. I receive video messages made to make fun of coronavirus.
3. People are making jokes related to the issues created by Coronavirus.
4. People are sending me videos made to acknowledge the efforts of health care workers.
5. People send me videos of their unusual home based activities during self-quarantine.
6. I receive messages or videos about the miseries of coronavirus patients.

Seeking and sharing COVID-19 information

1. I use social media to keep myself updated on current situation of the outbreak of covid-19.
2. I seek and share information on social media about the availability of face mask in my locality.
3. I seek and share information on social media about appropriate technique to use face mask.
4. I seek and share information on social media about the availability of hand sanitizers in my locality.
5. I use social media pages to take advice from the doctors in case of not feeling good.
6. I seek and share information on social media about preventive measures required to prevent myself from corona virus.
7. 1 seek and share information on social media given by the government to avoid the spread of coronavirus.

Section Three

Self-Efficacy of COVID-19

1. I can avoid COVID-19 infection
2. I can figure out how to avoid COVID-19 infection
3. I can recover even if I contract COVID-19
4. I am fully informed about COVID-19

Perceived Threat

1. Compared to other diseases COVID-19 is the most dangerous
2. COVID-19 is a serious threat to me and my family
3. The COVID-19 is serious risk that threatens the survival of humans
4. I have a high possibility of contracting COVID-19
5. Compared to others I am highly likely to contract COVID-19

Perceived Susceptibility

1. COVID-19 could happen to me
2. COVID-19 infection could happen to my family
3. COVID-19 infection could happen to my neighbors and friends
4. COVID-19 infection could happen any time to anyone, even a healthy individual

Perceived Severity

1. COVID-19 causes death quickly
2. Many people can die from COVID-19
3. A person who contracts COVID-19 will die if not treated
4. COVID-19 coronavirus is fatal

Section Four

COVID-19 Preventive Behavior

Handwashing

1. I use hand sanitizer before and after contact with high touch surfaces and objects
2. I wash my hands with soap and water for twenty seconds before having food
3. I was my hands with soap and water for twenty seconds before making food
4. I wash my hands with soap and water for twenty seconds whenever I feel my hands are dirty
5. I wash my hands with soap and water for twenty seconds after using the washroom

Cough Etiquette

1. I cover my mouth and nose every time I cough or sneeze
2. If no tissue is available I cough or sneeze in my arm
3. I put my used tissue in a covered waste basket

Other Basic Preventive Measures

1. I avoid hand shaking with people
2. I keep social distancing while moving out
3. I avoid to meet with sick people
4. I avoid going out unnecessarily
5. I avoid touching eyes, nose and mouth
